# Differentially Methylated Ultra-Conserved Regions Uc160 and Uc283 in Adenomas and Adenocarcinomas Are Associated with Overall Survival of Colorectal Cancer Patients

**DOI:** 10.3390/cancers12040895

**Published:** 2020-04-07

**Authors:** Anastasia E. Kottorou, Foteinos-Ioannis D. Dimitrakopoulos, Anna G. Antonacopoulou, Georgia Diamantopoulou, Dimitrios Tsoumas, Angelos Koutras, Thomas Makatsoris, Michalis Stavropoulos, Konstantinos C. Thomopoulos, Alicia Hulbert, Vassiliki Tzelepi, Haralabos P. Kalofonos

**Affiliations:** 1 Clinical and Molecular Oncology Laboratory, Division of Oncology, Medical School, University of Patras, 26504 Rio, Greece; kottorou@upatras.gr (A.E.K.); fodimitrak@yahoo.gr (F.-I.D.D.); antonac@gmail.com (A.G.A.); tsoumas2@gmail.com (D.T.); angkoutr@otenet.gr (A.K.); maktom@yahoo.com (T.M.); 2 Division of Gastroenterology, University Hospital of Patras, 26504 Rio, Greece; geodiamant@hotmail.com (G.D.); kxthomo@hotmail.com (K.C.T.); 3 Division of Oncology, Department of Internal Medicine, University Hospital of Patras, 26504 Rio, Greece; 4 Department of Surgery, Medical School, University of Patras, 26504 Rio, Greece; mstavrop@upatras.gr; 5 Cancer Center, University of Illinois at Chicago School of Medicine, Chicago, IL 60612, USA; ahulbert@uic.edu; 6 Department of Surgery, University of Illinois at Chicago School of Medicine, Chicago, IL 60612, USA; 7 Department of Pathology, Medical School, University of Patras, 26504 Rio, Greece; vtzelepi@yahoo.com

**Keywords:** transcribed-ultra conserved regions, colorectal cancer, adenomas, methylation, prognostic biomarkers

## Abstract

Deregulation of the transcribed ultra-conserved regions (T-UCRs) Uc160, Uc283, and Uc346 has been reported in colorectal cancer (CRC) recently. Here, we investigated promoter methylation of these T-UCRs during the adenoma–carcinoma sequence and their clinical significance in CRC patients. Methylation levels were assessed in CRC, adenomas, infiltrated lymph nodes, and metastatic tissue specimens. In situ hybridization was performed in representative tissue specimens. T-UCRs expression levels were also evaluated in HT-29 colon cancer cells before and after the acquired resistance to 5-fluorouracil (5-FU) and oxaliplatin. A gradual increase in T-UCRs methylation levels from hyperplastic polyps to adenomas and to in situ carcinomas (ISC) and a gradual decrease from ISC to infiltrative and metastatic carcinomas was observed (*p* < 0.001 for Uc160 and Uc283, *p* = 0.018 for Uc346). Uc160 and Uc283 methylation was associated with the grade of dysplasia in adenoma specimens (*p* = 0.034 and *p* = 0.019, respectively). Furthermore, higher Uc160 methylation, mainly in stage III and IV patients, was related to improved overall survival (OS) in univariate (*p* = 0.009; HR, 0.366) and multivariate analysis (*p* = 0.005; HR, 0.240). Similarly, higher methylation of Uc283 was associated with longer OS (*p* = 0.030). Finally, T-UCRs expression was significantly reduced in HT-29 cells after resistance to chemotherapy. This study suggests that promoter methylation of Uc160, Uc283, and Uc346 is altered during CRC development and that Uc160 and Uc283 methylation may have prognostic significance for CRC patients.

## 1. Introduction

Colorectal cancer (CRC) is the third leading cause of cancer-related deaths in men and women worldwide [1]. The classical pathway of colorectal tumorigenesis is characterized by the adenoma–carcinoma sequence, which can last decades, and it is determined by specific molecular pathways, including genetic alterations of tumor suppressor genes [2]. In this context, classical adenomas are precursor lesions, which are characterized from proliferative and dysplastic epithelial cells with increased potential for malignant transformation [2]. Apart from genetic alterations, the accumulation of epigenetic alterations plays an important role in CRC initiation and progression, while aberrant methylation of promoters leads to silencing of tumor suppressor genes or DNA damage repair genes [3,4].

Ultra-conserved regions (UCRs) are 481 genomic regions longer than 200 bp, which are 100% conserved among human, mouse, and rat genomes [5]. Some of them are transcribed into RNA (transcribed UCRs, T-UCRs) in a tissue-specific pattern, the transcription of which is deregulated in various types of cancer [6]. Transcriptional deregulation of T-UCRs has been attributed mainly to hypermethylation of the promoters, as well as to their interaction with microRNAs (miRNAs) [7,8]. During the last decade, increasingly more publications implicate T-UCRs in human diseases and especially in cancer [9]. Indeed, deregulation of specific T-UCRs has been documented in many malignancies, such as hepatocellular carcinoma [10,11], pancreatic [12], prostate [13,14], neuroblastoma [15], lung [16,17], gastric [18], leukemia [19,20] and CRC [19,20,21,22], while they have been associated with the clinical outcome of cancer patients [15].

In particular, T-UCRs Uc160, Uc283, and Uc346 are silenced in colon cancer cells due to specific CpG island hypermethylation, while DNA hypomethylation can reverse this effect [22]. In a previous study, we showed that methylation levels of Uc160, Uc283, and Uc346 are higher in CRC tissues compared to adjacent non-neoplastic tissues and that their expression levels follow an inverse pattern compared to methylation [23]. Moreover, proliferation and motility studies of Uc160 and Uc346 in colon cancer cell lines suggested a tumor-promoting role in CRC progression. Surprisingly, methylation levels of Uc160, Uc283, and Uc346, which had been assessed in a very small number (*n* = 6) of adenomas, were higher than normal and similar or higher compared to adenocarcinoma tissues, which motivated us to investigate this observation further [23].

Against this background, the aim of this study was to investigate our hypothesis that methylation of Uc160, Uc283, and Uc346 is associated with the transition of pre-malignant lesions to in situ, infiltrative, and metastatic tumors as well as with the clinical outcome of CRC patients.

## 2. Results

### 2.1. T-UCRs Methylation Levels Differ between Benign and Malignant Tumors

**Figure 1:**
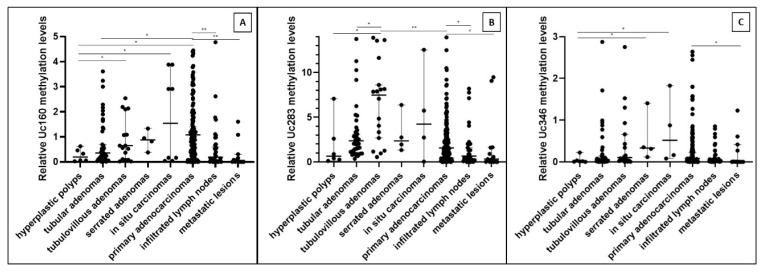
Relative tissue methylation levels of Uc160 (**A**), Uc283 (**B**), and Uc346 (**C**) in benign and malignant lesions and metastatic lesions (hyperplastic polyps, different types of adenomas, adenocarcinomas, infiltrated lymph nodes, and metastatic lesions; median values with 95% CI). * *p* < 0.05, ** *p* < 0.001.

T-UCR methylation analysis across the studied benign and malignant tissues revealed statistically significant differences following an almost “bell-shaped” pattern (*p* < 0.001 for Uc160, Figure 1A; *p* < 0.001 for Uc283, Figure 1Β; and *p* = 0.018 for Uc346, Figure 1C). Methylation levels of Uc160 and Uc346 gradually increased from hyperplastic polyps to adenomas and to in situ carcinomas, while in infiltrative adenocarcinomas methylation levels decreased, but they were still elevated compared to hyperplastic polyps (Figure 1). In particular, for Uc160 and Uc346, the highest methylation levels were observed in in situ adenocarcinomas, while for Uc283 in tubulovillous adenomas (Figure 1A–C). Moreover, lower methylation levels of Uc160 were observed in hyperplastic polyps compared to tubulovillous (*p* = 0.044) and serrated adenomas (*p* = 0.038), while lower levels of methylation were also observed between hyperplastic polyps and tubular adenomas compared to infiltrative adenocarcinomas (*p* = 0.011 and *p* = 0.002, respectively). In addition, the methylation levels of Uc283 in hyperplastic polyps were lower compared to tubulovillous adenomas (*p* = 0.006), as well as in tubular compared to tubulovillous adenomas (*p* = 0.019). Interestingly, tubulovillous adenomas were more methylated for Uc283 compared to adenocarcinomas (*p* = 0.001). Similarly, lower Uc346 methylation levels were observed in hyperplastic polyps in comparison with serrated adenomas (*p* = 0.019) as well as with in situ carcinomas (*p* = 0.038).

### 2.2. T-UCRs Methylation Levels in Benign Tumors Are Dysplasia Dependent

**Figure 2:**
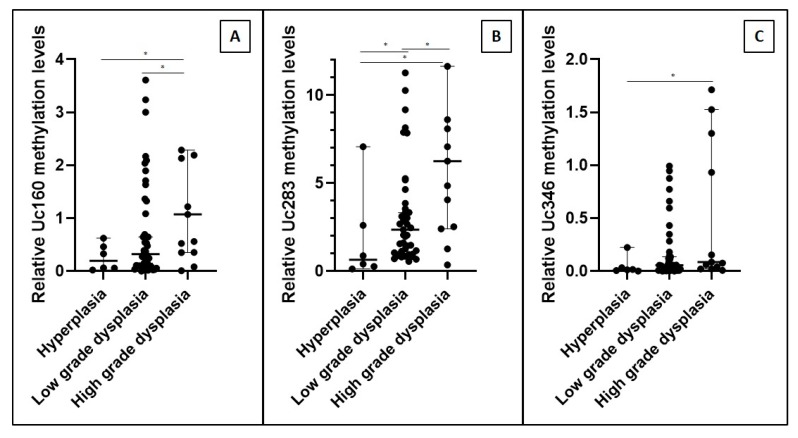
Relative tissue methylation levels of Uc160 (**A**), Uc283 (**B**), and Uc346 (**C**) in hyperplastic polyps and adenomas regarding the grade of dysplasia (median values with 95% CI). * *p* < 0.05.

Another intriguing finding was that methylation levels of the studied T-UCRs gradually increased depending on the grade of dysplasia (*p* = 0.034 for Uc160, *p* = 0.019 for Uc283, and *p* = 0.060 for Uc346, Figure 2). More specifically, adenomas with higher grade dysplasia were more methylated compared to hyperplastic polyps (*p* = 0.024 for Uc160, *p* = 0.032 for Uc283, and *p* = 0.024 for Uc346) and to adenomas with a low grade of dysplasia (*p* = 0.029 for Uc160, *p* = 0.050 for Uc283, and *p* = 0.148 for Uc346).

### 2.3. More Methylated T-UCRs in Adenocarcinomas Compared to Adenomas, Infiltrated Lymph Nodes, and Metastatic Lesions in the Same Patient

**Figure 3:**
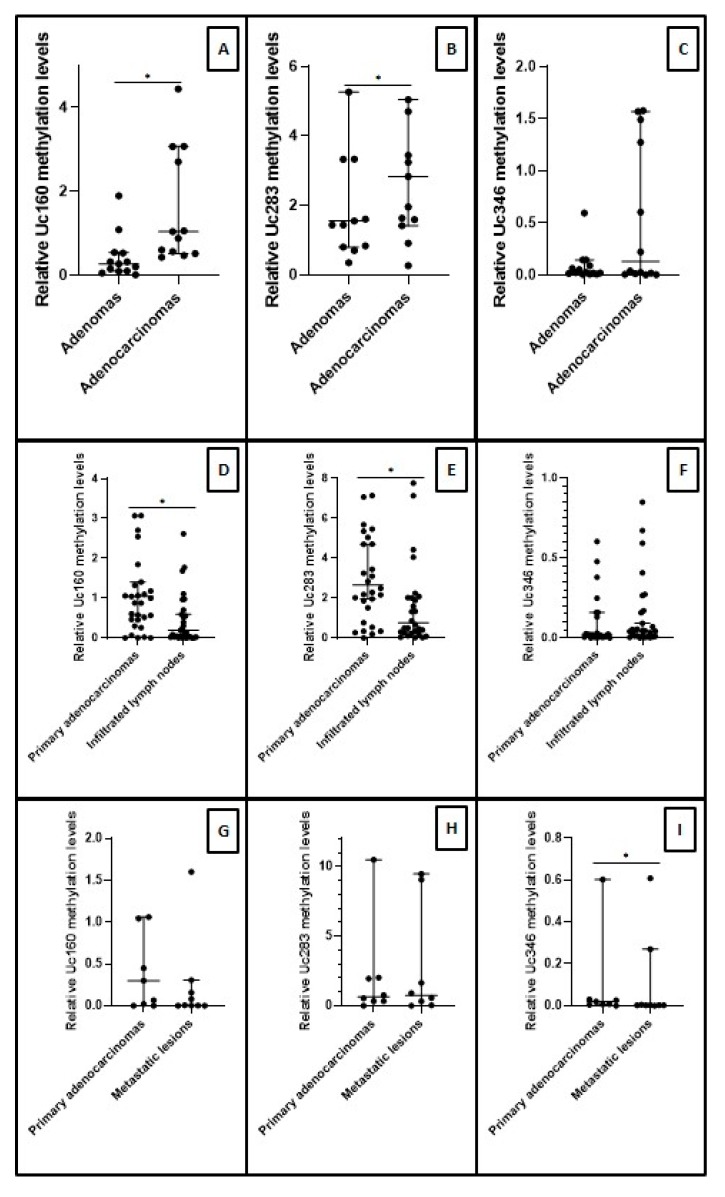
Relative tissue methylation levels of Uc160, Uc283, and Uc346 in primary tumors, in relation to paired adenomas, infiltrated lymph nodes, or metastatic lesions from the same patient. (**A**–**C**): methylation levels of Uc160 (**A**), Uc283 (**B**), and Uc346 (**C**) in adenomas and primary tumors; (**D**–**F**): methylation levels of Uc160 (**D**), Uc283 (**E**), and Uc346 (**F**) in primary tumors and infiltrated lymph nodes; and (**G**–**I**): methylation levels of Uc160 (**G**), Uc283 (**H**), and Uc346 (**I**) in primary tumors and metastatic lesions (median values with 95% CI).* *p* < 0.05.

The next step was to answer the question whether the methylation levels of Uc160, Uc283, and Uc346 differed in CRC patients bearing concurrent adenomas. Similarly to the above findings, T-UCR methylation levels in this subgroup of patients (*n* = 18) were higher in adenocarcinomas measured against adenomas (*p* = 0.001 for Uc160, Figure 3A; *p* = 0.048 for Uc283, Figure 3B), although this difference did not reach statistical significance for Uc346 (*p* = 0.064, Figure 3C). Additionally, primary adenocarcinomas had higher methylation levels of Uc160 and Uc283 compared to infiltrated lymph nodes (*p* = 0.006, Figure 3D and *p* = 0.023, Figure 3E, respectively), while such a difference was not observed for Uc346 (Figure 3F). Similarly, metastatic lesions had lower methylation levels of T-UCRs (Figure 3G–I) compared to adenocarcinomas, although only for Uc346 this difference was statistically significant (*p* = 0.038, Figure 3I).

The methylation levels of T-UCRs in adenomas tissue specimens were also compared between individuals with adenomas and CRC patients with a coexistence of adenomas. Interestingly, no statistically significant difference was revealed when all types of adenomas were analyzed (*p* = 0.829 for Uc160, *p* = 0.974 for Uc283, and *p* = 0.419 for Uc346, Figure S1A–C), or for low (*p* = 0.499 for Uc160, *p* = 0.937 for Uc283, and *p* = 0.937 for Uc346, Figure S1D–F), nor for high dysplasia adenomas (*p* = 0.214 for Uc160, *p* = 0.808 for Uc283, and *p* = 0.109 for Uc346, Figure S1G–I).

### 2.4. Gradual Decrease of T-UCRs Methylation from Primary to Metastatic Lesions

Unexpectedly, while methylation levels of T-UCRs gradually increased from hyperplastic polyps to adenomas and to in situ adenocarcinomas, an inverse pattern was observed from primary adenocarcinomas to infiltrated regional lymph nodes and metastases (Figure 1). The lowest methylation levels for all T-UCRs were observed in distant metastases and were significantly different compared to primary tumors (*p* < 0.001 for Uc160, Figure 1A; *p* = 0.020 for Uc283, Figure 1B; and *p* = 0.020 for Uc346, Figure 1C). Moreover, primary adenocarcinomas were also more methylated for Uc160 and Uc283 than infiltrated lymph nodes (*p* < 0.001 and *p* = 0.021, respectively).

### 2.5. T-UCRs Expression in Adenomas, Primary Tumor, and Metastatic Lesion

In order to confirm that methylation alterations among different types of tissues are also reflected in the expression level, in situ hybridization for Uc160, Uc283, and Uc346 was performed on representative tissue specimens from non-neoplastic adjacent tissue, hyperplastic polyps, adenomas, carcinomas, infiltrated lymph nodes, and distant metastatic lesions.

**Figure 4:**
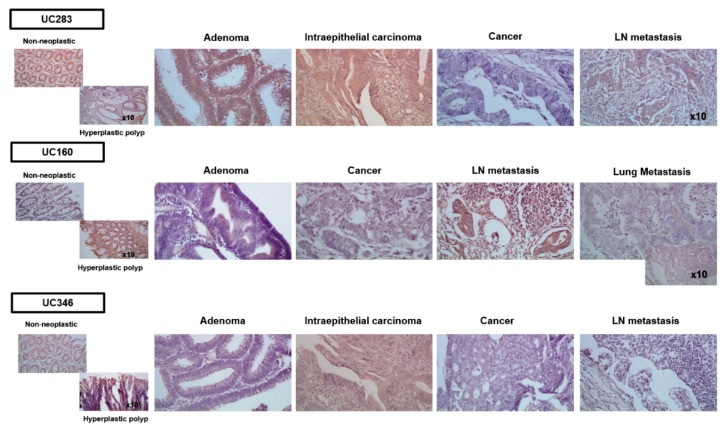
Microphotographs showing Uc160, Uc283, and Uc346 expression in non-neoplastic tissues, hyperplastic polyps, cancerous tissues, and metastatic lesions. LN: lymph node.

Generally, staining was seen mainly in the cytoplasm of epithelial cells, while stromal, endothelial, muscle, and immune cells were also positive in all three T-UCRs. For Uc160, intense staining was observed in hyperplastic polyps and in infiltrated lymph nodes and in adenomas, while adenoma with intramucosal carcinoma displayed faint staining. Faint staining was also observed in invasive carcinomas, while in non-neoplastic epithelium, the staining was faint to moderate (Figure 4). For Uc283, hyperplastic polyps and non-neoplastic epithelium displayed intense staining, while adenomas, carcinomas, and infiltrated lymph nodes had moderate staining (Figure 4). For Uc346, moderate staining was observed in hyperplastic polyps and non-neoplastic epithelium, while faint staining was observed in adenomas and infiltrating lymph nodes and faint to absent staining in carcinomas (Figure 4).

### 2.6. Methylation of Uc160 Is Associated with Improved Overall Survival

**Figure 5:**
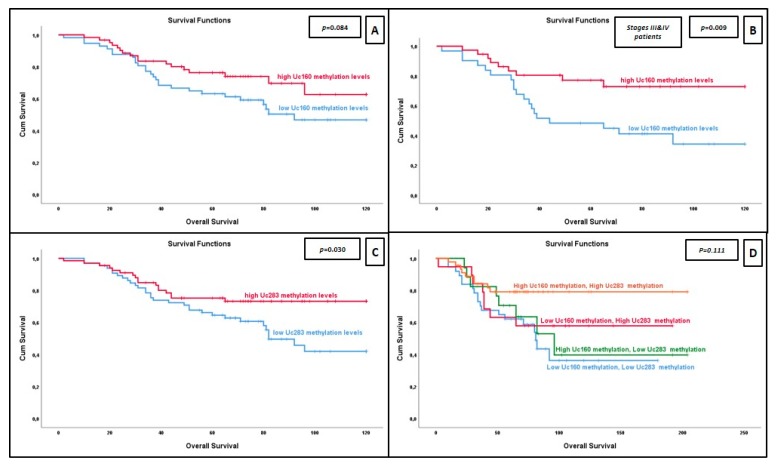
Kaplan–Meier curves for overall survival with regard to relative methylation levels of (**A**) Uc160 for all patients, (**B**) Uc160 for stages III and IV patients, (**C**) Uc283 and (**D**) Uc160 and Uc283 combination (high and low methylation levels determined by median values).

Among all CRC patients, higher methylation of Uc160 was associated with improved overall survival (OS), although this relation did not reach statistical significance (*p* = 0.084, Figure 5A). Additionally, the same trend was observed in patients older than 66 years (*p* = 0.074, Figure S2A). More interestingly, when infiltration of regional lymph nodes (LNs) was taken into account, patients with infiltrated LNs and higher methylation of Uc160 had improved OS compared to patients with lower methylation (*p* = 0.012, HR = 0.379; 95% CI, 0.171–0.839, Figure S2B). A similar association was not observed in patients with no infiltrated regional lymph nodes (*p* = 0.367). Further analysis by stage stratification revealed a prognostic value for methylation of Uc160 only for patients of stages III and IV (*p* = 0.009; HR, 0.366; 95% CI, 0.165–0.809, Figure 5B). Prognostic significance for OS persisted in multivariate analysis adjusted for age, tumor position, and tumor size (*p* = 0.005; HR, 0.240; 95% CI, 0.088–0.653).

### 2.7. Improved Survival for Patients with More Methylated Uc283 but Not for Uc346

Methylation of Uc283 was also associated with OS, with patients with higher methylation having longer survival (*p* = 0.030, Figure 5C). Further stratification with disease stage showed that stage III and IV patients with higher methylation levels of Uc283 had improved survival, although this relation did not reach statistical significance (*p* = 0.053, Figure S2C).

When the methylation levels of Uc160 were combined with the methylation levels of Uc283 (median values were used as cut-off points), patients with high methylation for both T-UCRs had improved OS compared to the other three groups, although this relation did not reach statistical significance (*p* = 0.111, for 4 tiers and *p* = 0.055 for 2 tiers, Figure 5D). On the other hand, the methylation levels of Uc346 had no association with OS, not even after stage stratification (*p* = 0.514, Figure S2D and *p* = 0.206, respectively). 

### 2.8. T-UCRs Expression in Chemotherapy-Resistant Colon Cancer Cells

**Figure 6:**
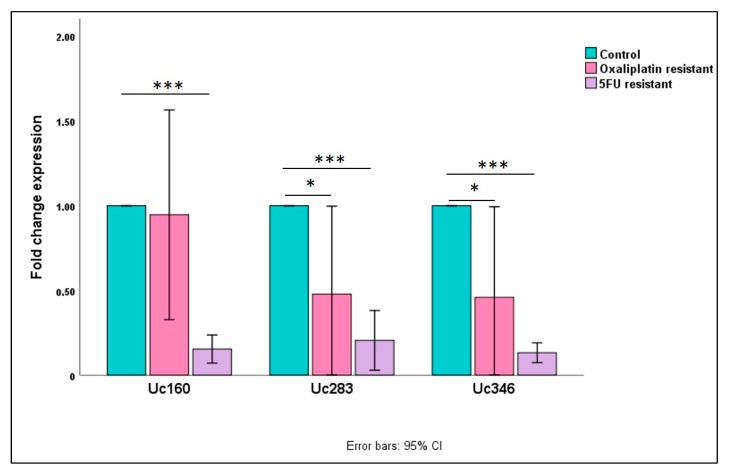
Fold change expression levels of Uc160, Uc283, and Uc346 in oxaliplatin and 5-fluorouracil (5-FU) resistant HT-29 cells compared to untreated cells (* *p* < 0.05, *** *p* < 0.0001).

Based on the differences we observed in survival rates depending on the disease stage and the T-UCRs methylation levels, we attempted to explore the impact of chemotherapy on T-UCRs expression levels on colon cancer cells. For that purpose, we assessed the Uc160, Uc283, and Uc346 expression levels in HT-29 cells before and after acquired resistance to 5-fluorouracil (5-FU) and oxaliplatin, chemotherapeutics that are widely used in CRC. Surprisingly, T-UCR expression levels were significantly lower in 5-FU-resistant HT-29 cells compared to untreated cells (*p* < 0.001 for all three T-UCRs, Figure 6). Similarly, the expression levels were reduced in oxaliplatin-resistant cells, a difference that was significant only for Uc283 and Uc346 (*p* = 0.049 and *p* = 0.048, respectively).

### 2.9. No Association of T-UCRs with Clinicopathological Parameters

When methylation levels of T-UCRs in adenocarcinomas were analyzed in relation to the clinicopathological parameters such as tumor differentiation (Figure S3A–C), lymph node infiltration (Figure S3D–F), distant metastasis (Figure S3G–I), disease stage (Figure S4A–C), and primary site (Figure S5A–C), no statistically significant difference was revealed.

## 3. Discussion

During the last decade, a growing number of studies have started to shed light on the role of T-UCRs in colon cancer [20,21,22,23]. Our group has demonstrated previously that the expression levels of T-UCRs Uc160, Uc283, and Uc346 were lower in CRC compared to non-malignant, tumor-adjacent tissues, while the methylation status of CpG islands in the promoters of specific T-UCRs displayed an inverse pattern [23]. In addition, overexpression of Uc160 and Uc346 was associated with increased proliferation and motility rates [23].

In this study, prompted by our previous work, we sought to clarify further the clinical significance of the methylation status of Uc160, Uc283, and Uc346 in individuals with different kinds of adenomas and patients with locally limited, locally advanced, or metastatic CRC. Particularly, the methylation levels of CpG islands in the promoter region of the three T-UCRs were assessed. Furthermore, their methylation levels were associated with OS, while expression of the studied T-UCRs was evaluated in HT-29 cells with regard to chemotherapy resistance.

One of the most interesting findings of the current study is the differential methylation levels of the investigated T-UCRs in benign and malignant tissues, which revealed an almost “bell-shaped” pattern. According to our knowledge, this is the first report in which the methylation levels of Uc160, Uc283, and Uc346 have been evaluated in multiple steps of the adenoma–carcinoma sequence. This finding shows that the methylation as well as expression of T-UCRs are variable during colon carcinogenesis in a step- and context-dependent manner. Further support to this observation comes from our findings that the methylation levels of T-UCRs in benign tumors are dysplasia dependent. This observation is compatible with the findings of Lujambio et al., who documented that these T-UCRs are demethylated and expressed in normal tissues, while they are hypermethylated and incur methylation-associated inactivation in cancerous tissues [22].

Another intriguing finding is the association of methylation of Uc160 with OS. Although this is the first study that has correlated the OS of CRC patients with methylation of Uc160, there are studies that have related the expression levels of other T-UCRs, such as Uc73 and Uc388, with the prognosis of CRC patients [21]. Additionally, Calin et al. documented that a combination of Uc160 expression with the expression of four other UCRs (Uc269, Uc215, Uc346, and Uc348) was able to differentiate between two main chronic lymphocytic leukemia (CLL) prognosis groups [20]. Notably, in gastric cancer, Pang et al. reported that Uc160 is significantly downregulated, while overexpression of Uc160 in SGC-7901 and AGS gastric cancer cells leads to the suppression of proliferation in vitro and in vivo [18]. In addition, our group have shown that Uc160 overexpression in HT-29, Caco-2, and DLD-1 colon cancer cells increases proliferation as well as cell motility [23].

The fact that high Uc160 methylation has a good prognostic impact while expression levels of Uc160 were significantly reduced in oxaliplatin- and 5-FU-resistant HT-29 cells may raise some concerns. This apparent contradiction may be explained taking into account the heterogeneity of CRC patients in our cohort, as well as the different impacts of methylation of Uc160 in the physical course of non-metastatic CRC patients vs. metastatic patients. In addition, metastatic or relapsed patients usually receive more than one line of systematic treatment and, in this context, we have no data regarding the role the studied T-UCRs with other chemotherapeutic, except 5-FU and oxaliplatin, or with biological regimens. In addition, it is well documented that miRNA-155, which directly targets Uc160 [20], is upregulated in colon cancer [24]. In turn, miRNA-155 overexpression induces chemoresistance of breast and colon cancer cells to cisplatin [25,26,27]. These findings are compatible with our observations and may explain at a molecular level our findings. Thus, it seems that the role of the studied T-UCRs is undoubtedly context dependent and treatment dependent, while their functionality warrants further investigation.

Another similar finding was the association of methylated Uc283 with the improvement of clinical outcome. Recently, Galasso et al. showed that Uc283 is highly expressed in pluripotent embryonic stem cells (ESCs) inducing pluripotency while it is overexpressed in gliomas and prostate cancer and less expressed in CRC [28]. In addition, a group led by Dr Esteller showed that Uc283 binds to pri-miRNA-195, leading to downregulation of mature miRNA-195 [29], a miRNA that is known to suppress metastasis and proliferation of CRC [30]. Furthermore, expression of miRNA-195 has been associated with chemoresistance in NSCLC [31] while, in gastric cancer patients, miRNA-195 overexpression augments chemotherapy sensitivity of cisplatin and prolongs OS and progression-free survival (PFS) [32]. These observations are in agreement with our results, which show a decrease in the expression of Uc283 in HT-29 cells, after the development of resistance in oxaliplatin and 5-FU chemotherapeutics.

Despite the clinically interesting results of this study, we should note some weak points. A limitation of our study is the lack of particular data from functional studies on the role of T-UCRs in adenomas and in CRC, while a larger cohort and an independent validation group would be an ideal next step in order to draw more robust conclusions.

## 4. Materials and Methods

### 4.1. Patients and Human Tissues

**Figure 7:**
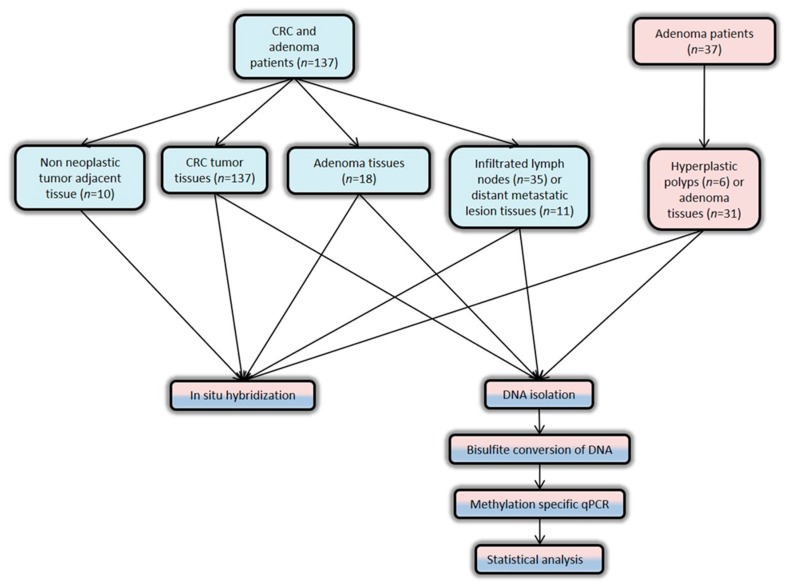
The workflow of the study.

The current study was approved by the Scientific Committee and the Committee on Research and Ethics of the University Hospital of Patras, Greece, prior to initiation of the study (approval #155-03/05/2012), and the ethical guidelines of the Helsinki Declaration (2013) were followed for the designation of this study [33]. The workflow of the study is shown in Figure 7.

In order to investigate our hypothesis, formalin-fixed paraffin-embedded (FFPE) tissue specimens were retrospectively selected and retrieved from the tissue archive of the Department of Pathology (University Hospital of Patras, Rio, Greece). The FFPE tissue specimens included 137 adenocarcinomas, 35 infiltrated lymph nodes, and 11 metastatic lesions from 137 patients with CRC; 49 adenomas from 31 patients with adenomas and 18 patients with simultaneous CRC and adenomas; as well as 6 hyperplastic polyps from 6 patients with adenomas (Table 1). Adenoma as well as cancerous (tumorous and infiltrated lymph node) specimens were removed during colonoscopies and surgeries, respectively. Three metachronous and four synchronous liver metastases, as well as four metachronous lung metastases were included in the current study. Diagnosis was determined based on the histological assessment. OS was assessed after a follow-up period of 120 months by using past medical histories, which were retrieved from the archive of the Division of Oncology (University Hospital of Patras, Rio, Greece), or through direct personal contact (via phone or in person).

### 4.2. DNA Isolation and Bisulfite Conversion of Genomic DNA

The “Methylation on Beads” (MOB) method was used for the genomic DNA isolation from FFPE tissue specimens and bisulfite conversion [34]. MOB is a single-tube method for the extraction and analysis of methylated DNA via the use of silica super magnetic beads and improves the extraction efficiency compared with conventional techniques [35,36]. After deparaffinization and lysis of the samples with proteinase K (Qiagen, Hilden, Germany) in 60 °C for 14 h, the lysate was mixed with 150 μL of magnesil paramagnetic particles (Promega, Madison, WI, USA). After purification of the beads with sequential washes and drying at 90 °C, the short protocol of the EZ DNA methylation Gold kit (Zymo Research, Irvine, CA, USA) was followed for the bisulfite conversion. Samples were incubated with bisulfite conversion reagent initially at 98 °C for 10 min and then at 64 °C for 2.5 h. After bisulfite conversion, DNA was desulfonated, washed, dried, eluted in 100 μL elution buffer, and stored at −20 °C.

### 4.3. DNA Methylation Analysis

Detection and quantification of Uc160, Uc283, and Uc346 methylation levels were performed using real-time quantitative Methylation Specific PCR (qMSP) with primers and probes previously designed and used by our group [23]. The primer and probe sequences are shown in Table S1 (Metabion International AG, Martinsried, Germany), while in Table S2 T-UCRs, the genomic positions according to Ucbase 2.0 are provided [37]. The total volume of the qPCR reactions was 20 μL and contained 2 μL of bisulfite converted DNA, 300 nM sense primer, 300 nM anti-sense primer, 100 nM probe, 10 nM reference dye (ThermoFischer Scientific, Waltham, MA, USA), 200 μM dNTPs (ThermoFischer Scientific, Waltham, MA, USA), and a single unit of Platinum Taq DNA Polymerase (ThermoFischer Scientific, Waltham, MA, USA) in 1X buffer containing 16.6 mM ammonium sulfate, 67 mM Tris pH8.8, 6.7 mM MgCl2, and 10 mM 2-mercaptoethanol [38]. All reactions were performed in triplicate and a methylated control (healthy donor blood DNA, in vitro methylated with SsI methylase, NEB Ipswich, MA and bisulfite converted) and unmethylated control (healthy donor blood DNA, bisulfite converted) were used, along with a no template control. Cycling conditions were the following: 95 °C for 10 min and 50 cycles at 95 °C for 30 s and at 60 °C for 1 min, 72 °C for 30 s with the MX3000P thermocycler (Stratagene, La Jolla, CA, USA). Relative methylation levels of T-UCRs were calculated using the LinRegPCR software [39]. Finally, β-actin was used for methylation levels’ normalization, with previously reported primers and probe [40]. The methylation analysis was performed blindly with respect to the specimen’s type and participants’ identities and data.

### 4.4. In Situ Hybridization

In situ hybridization of Uc160, Uc283, and Uc346 was performed on FFPE tissue specimens from CRC, non-neoplastic adjacent tissues, adenomas, infiltrated lymph nodes, and metastatic lesions as described elsewhere [41]. Tissue sections of 4μm were deparaffinized, rehydrated, and pretreated with proteinase K (Agilent, Santa Clara, CA, USA) for 20 min. A biotinylated Locked Nucleic Acid probe (Qiagen, Hilden, Germany) was used for the detection of the transcripts, in 1 nM concentration for Uc160 and Uc283 and 10 nM concentration for Uc346 in Enzo ISH buffer (Exiqon). Hybridization was performed at 37 °C for 2 h. A Dako GenPoint tyramide signal amplification system for biotinylated probes was used for the amplification of biotin detection, and the signal was finally developed with DAB chromogenic indicator.

### 4.5. Cell Line and Treatments

The human CRC cell line HT-29 was purchased from the American Type Culture Collection (ATCC, Manassas, VA, USA). HT-29 cells were cultured in RPMI 1640 medium with 100 μg/mL penicillin G/streptomycin, 2.5 μg/mL amphotericin B, 50 μg/mL gentamycin, and 10% fetal bovine serum (FBS). 5-FU and oxaliplatin were purchased from Teva Pharmaceuticals Hellas S.A. Resistant HT-29 cells to 5-FU and oxaliplatin were generated as described elsewhere [42]. Briefly, HT-29 cells were cultured with 5-FU or oxaliplatin at a concentration equal to 1/5 of the half maximal inhibitory concentration (IC50) value and the procedure was repeated until the final IC50 value was at least 5-fold greater than the IC50 value of the parental HT29 cells. Control cells were cultured with DMSO only. T-UCRs expression levels assessment was performed as described in our previous work [23].

### 4.6. Statistical Analysis

IBM SPSS Statistics for Windows, Version 21.0 (IBM Corp, Armonk, NY, USA) and GraphPad Prism 8 were used for all the statistical analyses. Kruskal–Wallis non-parametric test was used for the intergroup comparisons of Uc160, Uc283, and Uc346 methylation levels with the types of the tissue. Wilcoxon paired samples test was used for comparisons of methylation levels in different tissues of the same patient. Mann–Whitney and Kruskal–Wallis non-parametric tests were used for the association of the methylation levels of T-UCRs with clinicopathological characteristics of the patients. The Kaplan–Meier curves and the log rank test were used for the estimation of survival rates and the prognostic significance of the T-UCRs was evaluated by Cox regression analysis, with the median values used as the cutoff point. Cell line expression before and after chemotherapy was compared with paired T test. For all comparisons, statistical significance was defined as *p* < 0.05.

## 5. Conclusions

In conclusion, we report here that T-UCRs Uc160, Uc283, and Uc346 methylation is altered during the process of transformation to colorectal adenoma and carcinoma. Moreover, methylation of Uc160 and Uc283 has a prognostic significance for CRC patients.

## Figures and Tables

**Table 1:** Demographic and clinicopathological characteristics of CRC (colorectal cancer) and adenoma patients.

Demographic and Clinicopathological Characteristics		Cancer Patients	Adenoma Patients
Gender	Male	81 (59.1%)	24 (77.4%)
	Female	56 (40.9%)	7 (22.6%)
Age Group	66≥	62 (45.3%)	20 (64.5%)
	>66	74 (54.0%)	11 (29%)
Adenoma Grade of Dysplasia	Low	13 (9.5%)	23 (74.2%)
	High	5 (3.6%)	8 (25.8%)
Adenoma Architectural patterns	Tubular	14 (10.2%)	17 (54.8%)
	Tubulovillous	3 (2.2%)	11 (35.5%)
	Serrated	1 (0.7%)	3 (9.7%)
Stage	In situ	4 (2.9%)	N/A
	I	1 (0.7%)	N/A
	II	49 (35.8%)	N/A
	III	68 (49.6%)	N/A
	IV	13 (9.5%)	N/A
Grade	I	17 (12.8%)	N/A
	II	102 (72.8%)	N/A
	III	9(6.6%)	N/A
Tumor Primary Site	Right Colon	42 (30.7%)	N/A
	Left Colon and Sigmoid	49 (35.8%)	N/A
	Rectum	43 (31.4%)	N/A
Adenoma Primary Site	Right Colon	9 (50%)	12 (38.7%)
	Left Colon and Sigmoid	4 (22.2%)	13 (41.9%)
	Rectum	5 (16.1%)	6 (19.4%)
Lymph Node Infiltration	No	54 (39.4%)	N/A
	Yes	80 (58.4%)	N/A
Distant Metastasis	No	119 (86.9%)	N/A
	Yes	13 (9.5%)	N/A

## Supplementary Materials

The following are available online at https://www.mdpi.com/2072-6694/12/4/895/s1, Figure S1: Relative adenoma methylation levels of Uc160, Uc283 and Uc346 in adenoma and CRC patients with coexistence of adenomas alongside with the adenocarcinoma, Figure S2: Kaplan-Meier curves for overall survival with regard to relative methylation levels of A) Uc160 for patients older than 66 years, B) Uc160 for patients with regional lymph nodes infiltration, C) Uc283 for stage III and IV patients and D) Uc346, Figure S3: Relative tumor methylation levels of Uc160, Uc283 and Uc346 in relation to tumor differentiation, lymph nodes infiltration and distant metastasis, Figure S4: Relative tumor methylation levels of Uc160 (A), Uc283 (B) and Uc346 (C) in relation to disease stage, Figure S5: Relative tumor methylation levels of Uc160 (A), Uc283 (B) and Uc346 (C) in relation to primary tumor location, Table S1: Primers and probes sequences and annealing temperatures for qMSP and in situ hybridization, Table S2: T-UCRs names and positions according to Ucbase 2.0 (http://ucbase.unimore.it/).
