# Supplementary material for: Differentially Methylated Ultra-Conserved Regions Uc160 and Uc283 in Adenomas and Adenocarcinomas Are Associated with Overall Survival of Colorectal Cancer Patients
Source: Cancers (Basel). 2020 Apr 7;12(4):895. doi: 10.3390/cancers12040895 (PMC7226527; doi:10.3390/cancers12040895)
Supplement: Supplementary file 1 [file cancers-12-00895-s001.pdf]

Supplementary Materials

Differentially Methylated Ultra-Conserved Regions Uc160 and Uc283 in Adenomas and Adenocarcinomas Are Associated with Overall Survival of Colorectal Cancer Patients

Anastasia E. Kottorou, Foteinos-Ioannis D. Dimitrakopoulos, Anna G. Antonacopoulou, Georgia Diamantopoulou, Dimitrios Tsoumas, Angelos Koutras, Thomas Makatsoris, Michalis Stavropoulos, Konstantinos C. Thomopoulos, Alicia Hulbert, Vassiliki Tzelepi and Haralabos P. Kalofonos

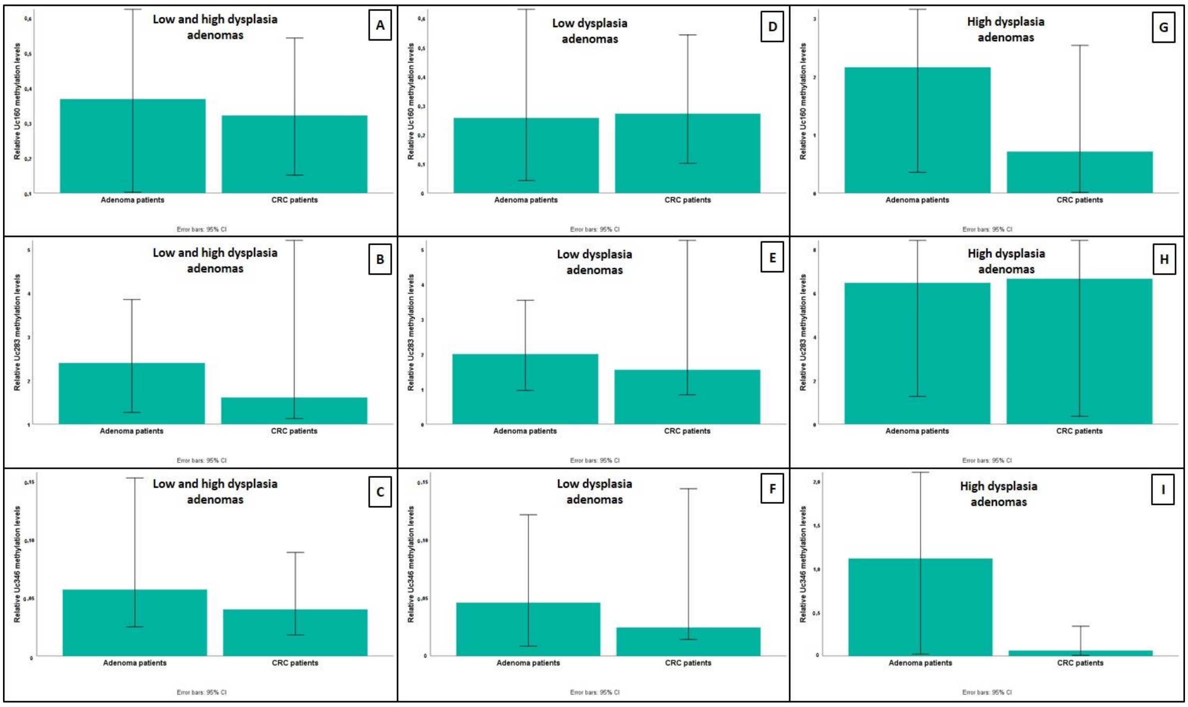

Figure S1. Relative adenoma methylation levels of Uc160, Uc283 and Uc346 in adenoma and CRC patients with coexistence of adenomas alongside with the adenocarcinoma; (**A**–**C**): methylation levels of Uc160 (**A**), Uc283 (**B**) and Uc346 (**C**) in low and high dysplasia adenomas; (**D**–**F**): methylation levels of Uc160 (**D**), Uc283 (**E**) and Uc346 (**F**) in low dysplasia adenomas; and (**G**–**I**): methylation levels of Uc160 (**G**), Uc283 (**H**) and Uc346 (**I**) in high dysplasia adenomas.

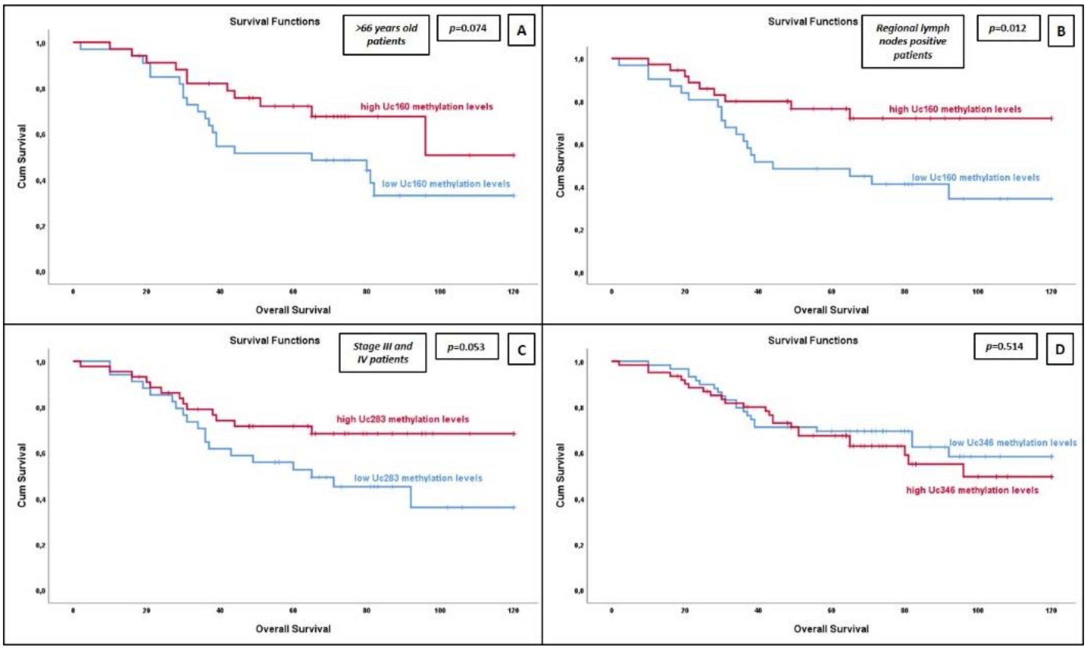

Figure S2. Kaplan-Meier curves for overall survival with regard to relative methylation levels of (**A**) Uc160 for patients older than 66 years, (**B**) Uc160 for patients with regional lymph nodes infiltration, (**C**) Uc283 for stage III and IV patients and (**D**) Uc346.

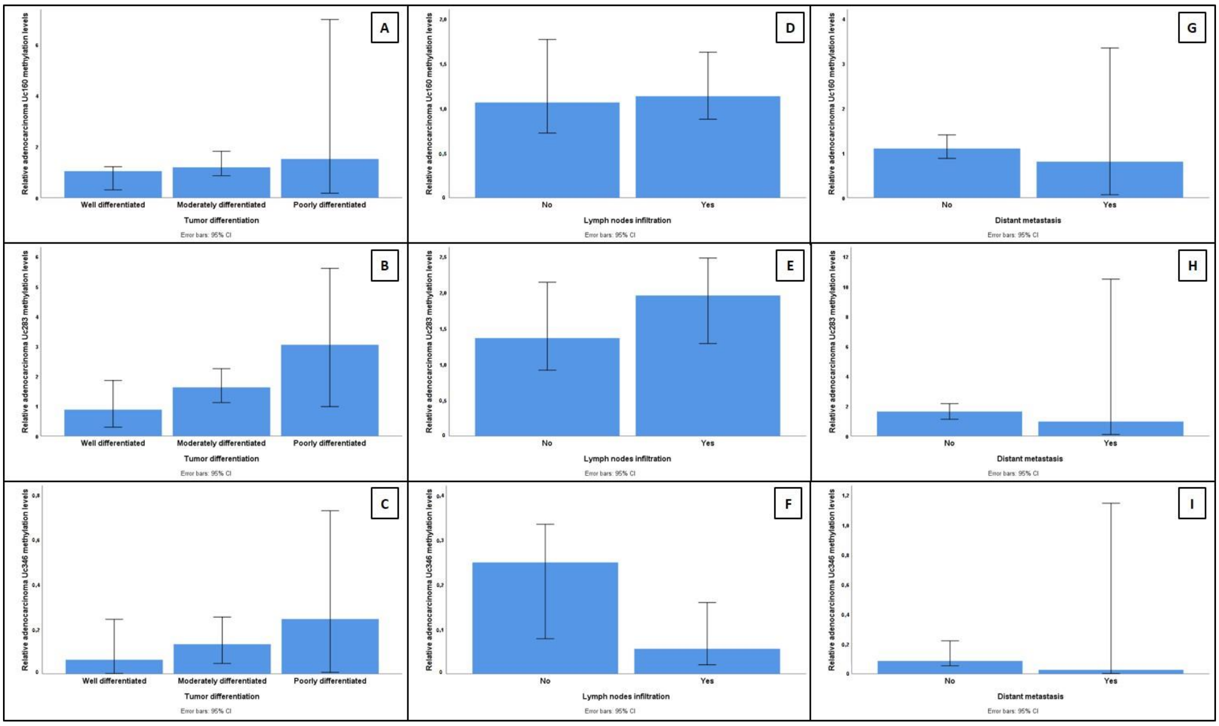

Figure S3. Relative tumor methylation levels of Uc160, Uc283 and Uc346 in relation to tumor differentiation, lymph nodes infiltration and distant metastasis; (**A**–**C**): methylation levels of Uc160 (**A**), Uc283 (**B**) and Uc346 (**C**) in relation with tumor differentiation; (**D**–**F**): methylation levels of Uc160 (**D**), Uc283 (**E**) and Uc346 (**F**) in relation with lymph nodes infiltration; and (**G**–**I**): methylation levels of Uc160 (**G**), Uc283 (**H**) and Uc346 (**I**) in relation with distant metastasis.

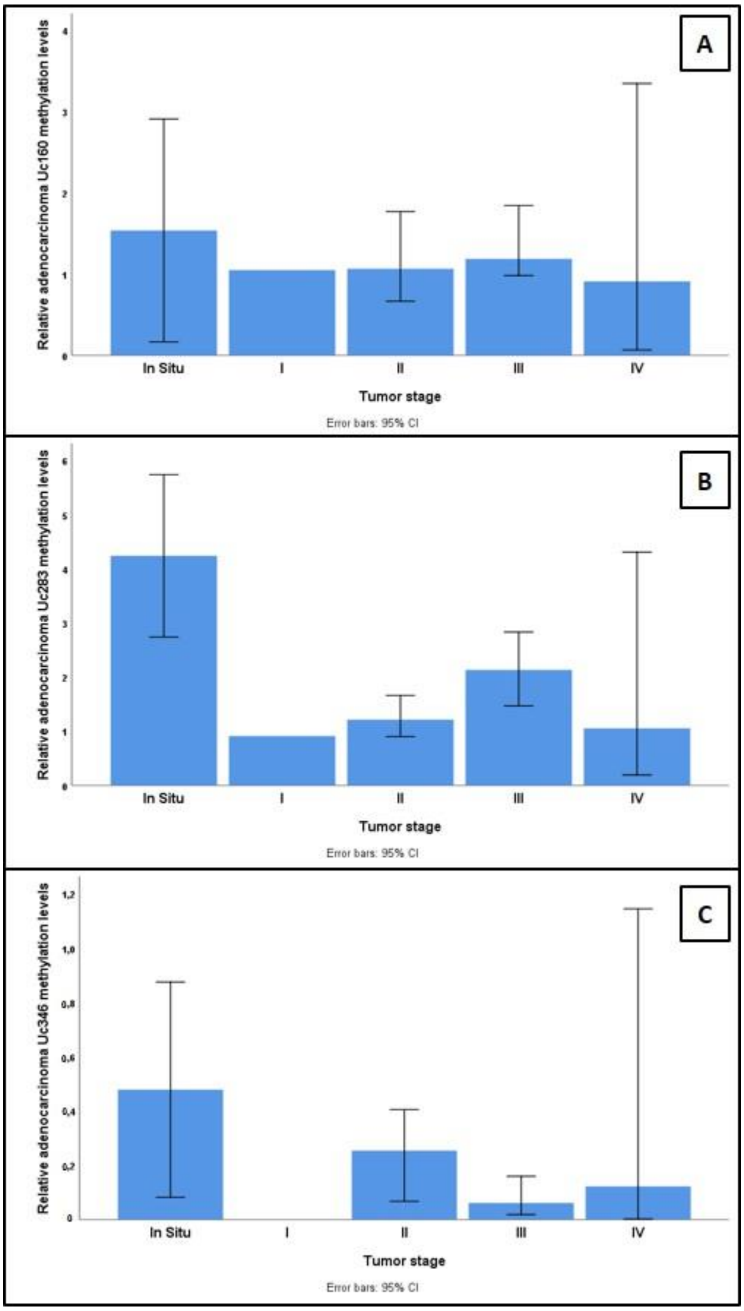

Figure S4. Relative tumor methylation levels of Uc160 (**A**), Uc283 (**B**) and Uc346 (**C**) in relation to disease stage.

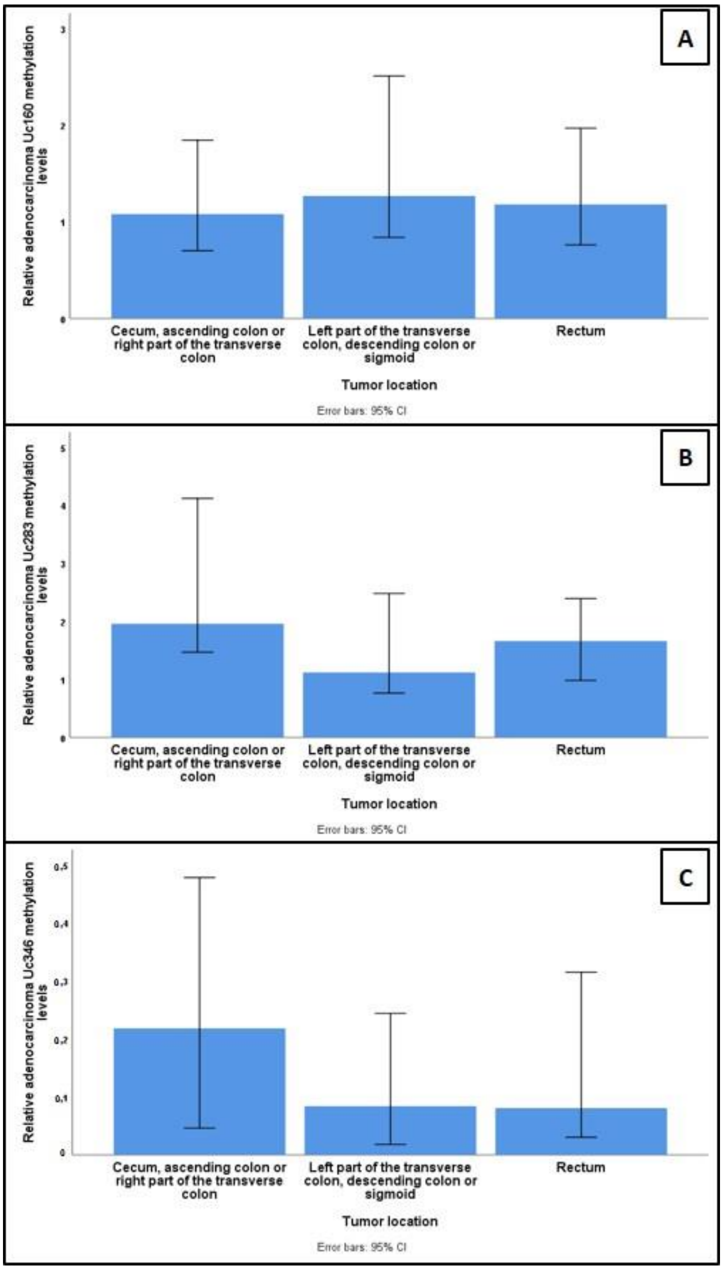

Figure S5. Relative tumor methylation levels of Uc160 (**A**), Uc283 (**B**) and Uc346 (**C**) in relation to primary tumor location.

Table S1. Primers and probes sequences and annealing temperatures for qMSP and in situ hybridization.

Primer/ Probe	Sequence (5′–3′)	Annealing Temperature (℃)
	Methylation assay (qMSP) primers and probes	
Uc160 FRW	ATTTTATTTATACGTTTATTCGGCG	
Uc160 REV	CCTACTCTAAACTCGTAAAACG	60
Uc160 probe	FAM/ T+CGGGG +CGTTG+CGGTTT /BHQ1	
Uc283 FRW	ATTCGTTTTCGGGATTTGTTAG	
Uc283 REV	CAAAACCACCGACTCCG	60
Uc283 probe	FAM/T+CGTTTTTTT+CGGGT +CGGTTGTT/BHQ1	
Uc346 FRW	ACGGCGTTAGGGATTTCG	
Uc346 REV	CGAATTACCCCGAATACTTTAACC	60
Uc346 probe	FAM/TTT +CGTTTT +CGT +CG+CGGT T/BHQ1	
b-actin FRW	GTGATGGAGGAGGTTTAGTAAGTT	
b-actin REV	CCAATAAAACCTACTCCTCCCTTAA	60
b-actin probe	FAM/ACCACCACCCAACACACAATAACAAACACA/BHQ1	
	In situ hybridization probes	
Uc160 ISH probe	ATCACAGATGTAGAGTATCAA/Bio	
Uc283 ISH probe	TTATGCGCCATTAGACTTGCT/Bio	
Uc346 ISH probe	TCTATTAGCATTAGCAAGCT/Bio	

(+C: LNA nucleotide).

Table S2. T-UCRs names and positions according to Ucbase 2.0 (http://ucbase.unimore.it/).

Uc Name	Chromosome	Start	End	Start hg18	End hg18	Upstream Gene Name	Downstream Gene Name
Uc160	5	77268844	77269165	77304600	77304921	AK128395	AP3B1
Uc283	10	50604757	50605033	50274763	50275039	AJ237663	ERCC6
Uc346	12	106976510	106976711	105500640	105500841	RPC2	RFX4

© 2020 by the authors. Licensee MDPI, Basel, Switzerland. This article is an open access article distributed under the terms and conditions of the Creative Commons Attribution (CC BY) license (http://creativecommons.org/licenses/by/4.0/).
